# From Nutrition to Innovation: Biomedical Applications of Egg Components

**DOI:** 10.3390/molecules30153260

**Published:** 2025-08-04

**Authors:** Amin Mohseni Ghalehghazi, Wen Zhong

**Affiliations:** Department of Biosystems Engineering, University of Manitoba, Winnipeg, MB R3P 0W8, Canada

**Keywords:** egg, biomaterials, biomedical applications

## Abstract

Valued for their nutritional content, eggs have recently gained attention as a versatile biomaterial owing to their biocompatibility, biodegradability, and unique structural and biochemical composition. This review highlights the biomedical potential of various egg components—eggshell, eggshell membrane, egg white, and egg yolk—and their applications in bone grafting, tissue engineering, wound healing, drug delivery, and biosensors. Eggshells serve as a natural, calcium-rich source for bone tissue engineering and regenerative medicine. The eggshell membrane, with its antimicrobial and structural properties, offers promise as a wound healing scaffold. Egg white, known for its gelation and film-forming capabilities, is utilized in hydrogel-based systems for drug delivery and biosensing. Egg yolk, rich in lipids and immunoglobulin Y (IgY) antibodies, is being explored for diagnostic and therapeutic applications. This review critically examines the advantages and limitations of each egg-derived component and outlines current research gaps, offering insights into future directions for the development of egg-based biomaterials in biomedical engineering.

## 1. Introduction

Eggs have been a staple in human diets for centuries, valued for their exceptional nutritional contents and versatility across a wide range of global cuisines. Beyond their culinary uses, eggs have also found applications in the nutraceutical industry because of their rich array of nutrients and bioactive compounds.

In recent years, the humble egg has gained attention as a promising biomaterial in the field of biomedical engineering. Its unique combination of properties, such as biocompatibility, biodegradability, and structural versatility, makes it an attractive candidate for various applications, including drug delivery, tissue engineering, and diagnostics platforms. This growing interest in eggs as biomaterials is driven by the demand for cost-effective and sustainable alternatives to traditional biomedical materials [1,2].

Eggs possess several key characteristics that make them highly suitable for biomedical applications. One of their primary advantages is their abundance and low cost, making them an accessible and economical choice for research and development. This affordability supports extensive experimentation and enhances the potential for scalable production. In addition, eggs are generally biocompatible and biodegradable, which helps minimize adverse biological reactions and reduces environmental impact [1]. They are rich in bioactive components that can promote cell proliferation, differentiation, and tissue regeneration. Notably, eggs have been found to stimulate angiogenesis, the formation of new blood vessels, which plays a crucial role in tissue regeneration and wound healing [3]. These compounds exhibit diverse functional properties, as summarized in Table 1.

**Figure 1:**
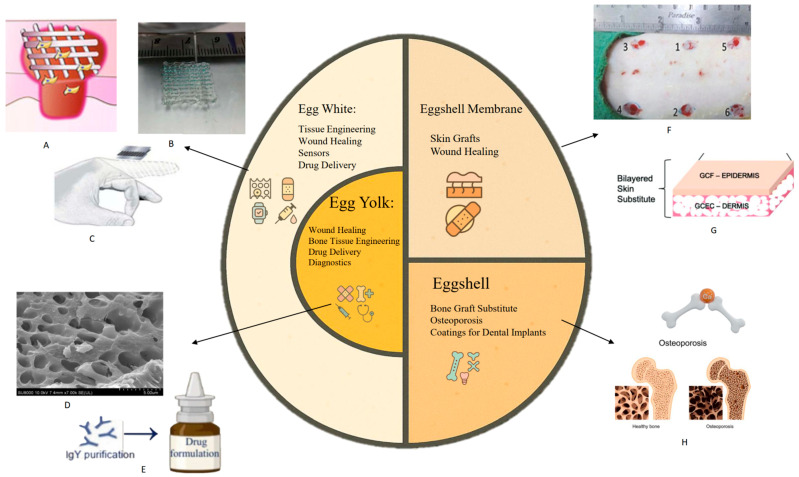
Biomedical applications of egg components. (**A**) 3D-printed egg white (EW) hydrogels for wound dressing [5]. (**B**) 3D printing of EW hydrogels [6]. (**C**) Motion sensor developed from Carbon nanotube (CNT)-EW hydrogels (Reproduced with permission from ref. [6] Copyright 2019, The Royal Society of Chemistry) [6]. (**D**) SEM image of egg yolk hydrogels [7]. (**E**) Purification of specific lgY for medical formulation (Reproduced with permission from ref. [8] Copyright 2023, Elsevier) [8]. (**F**) Bioglass wound dressing coated with eggshell membranes (Reproduced with permission from ref. [9] Copyright 2023, Elsevier) [9]. (**G**) Skin substitute from eggshell membrane crosslinked gelatin–chitosan cryogels (Reproduced with permission from ref. [10] Copyright 2021, The Royal Society of Chemistry) [10]. (**H**) Bone graft substitute, osteoporosis, coatings for dental implants.

Compared to traditional biomaterials derived from mammals, eggs offer a more sustainable and ethically sourced alternatives, addressing concerns related to animal welfare and environmental sustainability in biomaterial production [4]. Moreover, eggs are easy to handle and process, allowing for efficient extraction and utilization of their various components across a range of biomedical applications, as depicted in Figure 1.

This paper explores the potential of eggs as a biomaterial in biomedical engineering. It examines the unique properties that make eggs suitable for applications such as drug delivery, tissue engineering, and diagnostics. Specific examples are provided to illustrate how different parts of eggs have been utilized, highlighting their respective advantages and limitations. Finally, the paper discusses the future potential of eggs in biomedical applications and identifies current gaps and challenges that need to be addressed to advance this emerging field.

**Table 1:** Bioactive Compounds Found in Eggs and Their Functional properties.

Bioactive Compound	Functional Properties
Lysozyme	Antibacterial [11,12], anti-cancer [13], antiviral [14], immunomodulation [15], antihypertensive [16]
Ovalbumin	Antibacterial [17,18], antioxidant [19], immunomodulation [20]
Ovotransferrin	Antibacterial [21], anti-inflammatory [22], antioxidant [23], antiviral [24], immunomodulation [25]
Ovomucin	Antibacterial [26], anti-cancer [27,28], anti-adhesive [29], antioxidant [30], antiviral [28], immunomodulation [31]
Avidin	Antibacterial [32]
Phosvitin	Antibacterial [33], anti-inflammatory [34], antioxidant [35]
IgY	Antibacterial [36], anti-cancer [37], antiviral [38], immunomodulation [39]
Phospholipids	Anti-inflammatory [40], antioxidant [41]
Lutein/Zeaxanthin	Anti-inflammatory, antioxidant [42]
High-density Lipoproteins	Anti-inflammatory, antioxidant [43]
Cystatin	Protease inhibition [44]
Ovoinhibitor	Antibacterial [45], protease inhibition [46]
Egg Yolk Hydrolysates	Antihypertensive [47]

## 2. Applications of Eggs in Biomedical Engineering

The suitability of eggs for biomedical applications stems from the unique properties of their individual components. Eggshells, primarily composed of calcium carbonate, are well-suited for bone regeneration and hydroxyapatite synthesis [48]. Eggshell membrane, with its fibrous network rich in collagen and hyaluronic acid, exhibits antibacterial and wound-healing properties [49]. Egg white, abundant in proteins such as lysozyme and ovalbumin, offers antibacterial, anti-inflammatory, and cell growth-promoting effects [50]. Meanwhile, egg yolk contains a variety of lipids, proteins, vitamins, and minerals [51], contributing to wound healing and bone tissue engineering [52]. An overview of the applications is shown in Table 2.

### 2.1. Eggshell

Eggshells, composed primarily of calcium carbonate, have been widely utilized in biomedical applications, particularly for bone regeneration. Typically, eggshells are processed into fine powder before being used in dietary supplements or as a component in biomaterials. The industrial preparation of eggshell powder generally involves washing, heat treatment in a furnace, and subsequent grinding. For biomedical applications, the required particle size is often more specific and varies depending on the intended use. For example, ball milling techniques have been used to produce nano-sized eggshell powders for advanced applications [53,54].

Finely ground eggshell powder functions as a bioactive material that can enhance bone mineral density and mitigate bone loss, making it a promising candidate for osteoporosis treatment [55]. In addition to calcium, eggshells naturally contain trace elements such as boron and strontium, which further enhances their nutritional value for bone health. Research suggests that eggshell calcium has higher bioavailability compared to commercial calcium carbonate, making it a suitable component for dietary supplements [56]. One study showed that supplementation with eggshell powder, in combination with magnesium and vitamin D3, significantly improved bone mineral density in patients [57].

Various methods have been developed to incorporate eggshell powder into biomaterials, including direct mixing with polymer matrices, sol–gel processing, and hydrothermal treatment.

Among these, direct mixing is a straightforward and cost-effective technique that involves the physical incorporation of fine eggshell powder into a polymer matrix to create a composite biomaterial. This method leverages the desirable properties of both components, such as the bioactivity and biocompatibility of eggshells and the mechanical and structural advantages of polymers. The process typically encompasses the preparation of eggshell powder and the polymer, followed by physical blending and subsequent consolidation into the desired shape. Common techniques include melt mixing, solution mixing, and hand layup.

In melt mixing, often used for thermoplastics, the polymer is heated until molten, after which the fillers are added and thoroughly blended [58]. In solution mixing, both the polymer and filler are dissolved in a common solvent, and then the solvent is evaporated to yield the final composite material [59]. Hand layup, another direct mixing method, is often employed in fiber-reinforced composites, where eggshells are hand layup which is usually used for where the eggshell powder is dispersed in a resin that impregnates the reinforcing fibers [60].

Overall, while direct mixing offers simplicity and scalability, achieving uniform dispersion of the filler, especially at the nanoscale, can be challenging and may affect the consistency of the final material properties [61].

Sol–gel processing is a wet chemical technique commonly employed to produce solid materials, often metal oxides, from small molecular precursors. When applied to eggshells, this method uses the calcium carbonate content as a starting material to synthesize calcium-based compounds such as calcium oxide, hydroxyapatite, or bioactive glasses. The process involves transforming monomers into a colloidal solution (sol), which gradually transitions into a gel network. Compared to traditional ceramic manufacturing techniques like solid-state sintering, sol–gel processing typically requires lower temperatures. This advantage is particularly beneficial for preserving organic components or preventing thermal degradation of sensitive materials. Additionally, sol–gel techniques offer precise control over particle morphology and size, allowing the production of nanoparticles, thin films, and porous scaffolds. The size, shape, and surface characteristics of the resulting materials can be tailored by adjusting the reaction parameters. Moreover, the molecular-level mixing of reactants ensures a high degree of chemical homogeneity in the final product, enhancing its performance in biomedical applications [62,63].

Hydrothermal treatment, in the context of biomaterials and eggshell powder, is a method that involves reacting eggshell powder with an aqueous solution under high temperature (typically 100–250 °C) and high pressure in a sealed system, such as an autoclave, to convert it into a desired biomaterial, most commonly hydroxyapatite. This technique utilizes the enhanced solubility and reactivity of precursors in superheated water to facilitate chemical transformations [63,64]. Compared to high-temperature calcination, hydrothermal treatment is often carried out at relatively lower temperatures, making it more energy-efficient and environmentally friendly [65]. By carefully controlling the reaction parameters such as temperature, pressure, duration, and reactant concentrations, the morphology, particle size, and crystallinity of the resulting hydroxyapatite can be finely tuned for specific biomedical applications [64]. Additionally, eggshell powder is incorporated into bio-epoxy composites and polyamide-based materials to improve mechanical properties, including tensile, flexural, and impact strength [66,67,68]. These composites have potential applications in structural biomaterials, such as dental and orthopedic implants, where enhanced load-bearing capacity is essential [69].

However, excessive loading of eggshell powder may adversely impact the flexural and tensile strength of the composite materials, underscoring the importance of optimizing filler content for balanced performance [70].

Eggshell powder is often combined with biopolymers such as polycaprolactone (PCL) or nanostructures like carbon nanotubes to develop hybrid scaffolds for bone tissue engineering [71,72]. In particular, the incorporation of eggshells improved the hydrophilicity and biocompatibility of the composite scaffolds, such as those composed of carbon nanotubes and eggshell fillers within a PCL matrix [71].

One of the most widely used materials derived from eggshells is hydroxyapatite (HAp). Eggshell-derived HAp scaffolds exhibit excellent osteoconductivity and bioactivity, making them well-suited for use as bone graft substitutes and coatings for dental implants [48]. These scaffolds feature a porous structure that supports the infiltration of osteogenic (bone-forming) cells and promotes vascularization, both of which are critical for effective new bone formation [49].

Recent studies have explored composite materials incorporating eggshell-derived HA with PCL, sodium alginate and egg whites to enhance scaffold biocompatibility. Results showed that scaffolds containing higher amounts of egg white and egg shell demonstrated improved biocompatible [73]. Additionally, carbon nanotubes have been integrated with HA to improve mechanical strength, making these composites suitable for load-bearing bone defects. These HA-based materials have shown promising results in enhancing osseointegration, reducing inflammation, and accelerating bone fracture healing [1].

### 2.2. Eggshell Membrane

Eggshell membrane (ESM) is a naturally occurring biopolymer with a fibrous structure composed of collagen, hyaluronic acid, and glycoproteins. Its architecture provides an excellent scaffold for cell attachment, proliferation, and differentiation, making it suitable for applications in wound healing, skin grafts, and tissue engineering [74]. This is attributed to the chemical similarity between eggshell membrane (ESM) and human bone, as both primarily consist of type I collagen, chondroitin sulfate, dermatan sulfate, hyaluronic acid, and elastin. Moreover, studies have shown that ESM improves calcium uptake in human cells, alleviates pain in patients with joint disorders, and reduces inflammation [75]. Processed eggshell membrane powder has been explored for its antibacterial and biocompatible properties in wound healing. Studies have shown that processed eggshell membrane powder significantly expedited wound closure [76].

Furthermore, hybrid scaffolds combining ESM with synthetic polymers, such as polycaprolactone (PCL), have demonstrated improved biocompatibility and antibacterial properties, making them promising candidates for wound dressing applications [77]. Owing to its excellent biocompatibility and low immunogenicity, ESM has also been explored as a bioactive component in polymeric scaffolds for enhanced bone regeneration. In one study, a PCL scaffold was coated with ESM by soaking it in an ESM solution followed by vacuum drying to remove the solvent. The ESM coating significantly promoted bone-forming cell differentiation and adhesion [78]. Eggshell membrane has been utilized I combination with PLGA to develop a biocompatible drug-incorporated bandage containing Fluorescein isothiocyanate-labeled Bovine Serum Albumin (FITC-BSA), leveraging its capacity to serve as a bioactive carrier for controlled drug release. This drug-incorporated bandage has been used in cornea wound healing [79].

### 2.3. Egg White

Egg white is widely recognized for its biocompatibility, biodegradability, and gelling properties, making it valuable for various biomedical applications, including tissue engineering, wound healing, and drug delivery [1]. Its capacity to encapsulate and release therapeutic agents in a controlled manner makes it particularly attractive as a drug carrier and component in drug delivery systems [80]. Additionally, egg white can serve as a biocompatible matrix for immobilizing enzymes or antibodies in biosensors, enabling the detection of various analytes [2].

Egg white proteins, such as ovalbumin and ovotransferrin, exhibit antioxidant activity, protecting cells from oxidative stress induced by free radicals [19]. This property is advantageous for wound healing and tissue regeneration. Additionally, egg white proteins can bind to growth factors, which are signaling molecules involved in regulating cell growth and differentiation [81]. Another notable feature of egg white is its transparency, which allows for easy visualization of cells and tissues within the gel matrix. This makes it particularly useful for in vitro studies and tissue engineering applications where monitoring cell behavior and tissue development is critical. For instance, egg white was employed as a substitute for extracellular matrix (ECM) in an angiogenesis assay. The egg white matrix demonstrated excellent compatibility with various human, mouse and rat cell types, supporting its utility in such biological evaluations [82]. Egg white hydrogels are gaining attention in biomedical applications due to their biocompatibility, biodegradability, bioactivity, mechanical tunability, and shear-thinning behavior, and low cost [6,83,84]. Their shear thinning property, where viscosity decreases with increasing shear stress, makes them particularly suitable for applications such as injectable drug delivery systems and 3D printing [85,86,87]. Additionally, various crosslinking methods, including physical [6], thermal [88], and enzymatic approaches [89], can be employed to tailor the mechanical and biological properties of egg white hydrogels for specific applications.

Egg white hydrogels have shown potential in wound healing by promoting tissue engraftment and stimulating angiogenesis [3]. Their three-dimensional structure and biocompatibility also support cell attachment, proliferation, and differentiation, making them promising scaffolds for tissue engineering and drug delivery systems [90]. For example, one study developed an albumin-based hydrogel composed of starch and albumin, which featured a porous, non-toxic structure suitable for various cell culture application [91]. Moreover, the inherent antibacterial properties of egg white, primarily due to its lysozyme content, further enhance its potential for biomedical use [92].

Egg white has also been explored for use in motion sensors due to its self-healing and conductive properties. These properties have been achieved by physically crosslinking egg white hydrogels with calcium chloride and Dulbecco’s Modified Eagle Medium (DMEM) and further enhanced by incorporating carbon nanotubes to improve electrical conductivity, making the hydrogel sufficient sensitive for detecting human motion and health signals [6]. The self-healing capacity, defined as the ability of a material to repair damage such as cracks from mechanical stress, is particularly advantageous in wearable and flexible electronics. Owing to its phase transition properties, egg white hydrogels have also been explored for applications in wearable electronics, human–machine interfaces, and clean energy harvesting [84].

In the realm of biosensing, egg white proteins have demonstrated potential as biocompatible and cost-effective matrices. In one study, egg white protein microparticles were used to create a 3D scaffold that accommodated glucose oxidase and redox molecules for glucose levels detection [93]. Another study developed a multifunctional hydrogel sensor composed of egg white, gelatin, and carboxymethyl cellulose. This hydrogel exhibited excellent strain, stress, and motion sensing capacities. The functional proteins in egg white contributed to self-healing and self-adhesive properties of the prepared hydrogel, making it suitable for next-generation bioelectronic devices [94].

Hybrid scaffolds combining egg white with biomaterials such as gelatin, chitosan, and silk fibroin have demonstrated improved mechanical strength and biocompatibility [95]. Gelatin is a protein derived from collagen and a key component of the extracellular matrix (ECM). It provides structural support and promotes cell adhesion and proliferation. When combined with EW, gelatin enhances the mechanical strength and stability of the hydrogel, making the hybrid hydrogel suitable for applications in wound healing and tissue engineering [95]. In one study, egg white and gelatin were mixed to form hydrocolloids that served as a 3D scaffold for cell studies within a microfluidic device. The microfluidic device contained a central chamber where the sterile-filtered hydrocolloids were introduced via microchannels. Additionally, two larger side media channels ran parallel and connected to the central chamber, providing continuous hydration and nutrient delivery to the cultured cells [96].

PCL has also been incorporated with egg white and gelatin to develop scaffold for tissue engineering applications. In one study, researchers demonstrated that adding 10% egg white to PCL/gelatin nanofibers significantly improved fibroblast culture efficiency, indicating enhanced cell adhesion and proliferation [97]. Alginate, a polysaccharide derived from brown algae, forms hydrogels in the presence of divalent cations like calcium, creating a three-dimensional structure for cell encapsulation and tissue regeneration. EW-alginate hydrogels have been investigated for applications such as drug delivery and cartilage regeneration. In this combination, EW contributes ECM-like proteins, while alginate provides excellent mechanical tunability through ionic crosslinking. This allows researchers to fine-tune properties such as porosity, stiffness and strength to meet specific biomedical needs [98].

Chitosan, a linear polysaccharide derived from chitin found in crustacean shells, possesses antibacterial properties and promotes wound healing. EW–chitosan hydrogels have shown promise in wound dressings and drug delivery systems. For instance, a hydrogel composed of hydroxypropyl chitosan and egg white showed promising properties for treating burn wounds [99].

Silk fibroin, a natural protein obtained from silkworms, is known for its excellent biocompatibility, mechanical strength, and slow degradation rate. Combining silk fibroin with EW improves the mechanical properties and structural stability of the resulting hydrogel, making it well-suited for tissue engineering and drug delivery applications [100].

In a recent study, carbon dots were incorporated into EW hydrogels, enhancing their integration with the surrounding tissue and aligning their degradation rate with the timeline of hair follicles regeneration [101].

Additionally, egg-based assays are commonly used to diagnose egg allergies in individuals. The allergenic properties of egg proteins are particularly relevant in diagnostics applications. Several major egg allergens have been identified, each with distinct structural and immunological characteristics [102].

### 2.4. Egg Yolk

Egg yolk contains a rich array of lipids, proteins, vitamins, and minerals, supporting its applications in wound healing and bone tissue engineering [52]. Notably, it is a source of immunoglobulin Y (IgY) [103], which has shown promise in both drug delivery and diagnostics. IgY antibodies, derived from egg yolk, have been investigated for the diagnosis and treatment of bacterial and viral infections. Because of their antibacterial properties, IgY has been incorporated into oral hygiene products such as toothpastes and mouthwash. For example, IgY preparations have been shown to stimulate the release of cytokines like TNF-α and IL-1β, which are involved in immune responses and inflammation. Compared to mammalian antibodies, IgY offers advantages such as non-invasive harvesting and potentially higher specificity in certain diagnostic applications [104]. Furthermore, egg yolk phospholipids are utilized in the formation of liposomes, which serve as effective drug carriers [105]. Phosvitin, a highly phosphorylated protein found in egg yolk, has also shown potential in combating osteoporosis. Studies suggested that phosvitin promotes osteoblast differentiation in a manner comparable to ascorbic acid, highlighting its promise as a therapeutic compound in bone regeneration and osteoporosis treatment [106].

### 2.5. Other Applications

Whole eggs have found diverse applications in the field of biomedical engineering. Chicken eggs, in particular, have been explored as bioreactors for producing protein-based therapeutics. Through genetic modification, researchers have engineered chickens to lay eggs containing specific drug proteins in their egg white, such as Interferon a2a, a treatment for cancer and hepatitis, and CSF1-Fc, a protein with potential applications regenerative medicine. This method offers a promising strategy to produce therapeutic proteins more cost-effectively and at larger scales [107].

Additionally, researchers have used fertilized chicken eggs as a model for cancer imaging studies and radiotracer development. The chorioallantoic membrane (CAM), a highly vascularized membrane in chicken eggs, provides an ideal environment for tumor growth and study. This approach enables the cultivation and imaging of tumors within seven days, providing a rapid, cost-effective and ethically sustainable alternative to traditional mammalian models for preclinical cancer research. The CAM model allows for the efficient assessment of tumor-targeting drugs and radiation therapies [108]. A similar approach has also been applied using ostrich egg, which offer a larger platform for certain experimental setups [109].

**Table 2:** Overview of biomedical applications of egg components.

Egg Component	Application	Key Components	Role of the Egg Components	Performance
Eggshell	Bone repair	PCL/ES powder/CNT [71]	Provides a source of hydroxyapatite, a key component of bone	Abundant, inexpensive, biocompatible, osteoconductive, brittle, potential immune response
	Used as a filler for improving strength	ES-derived Calcium Oxide/CNT [110]	Source of calcium, environmentally friendly	Improved tensile strength, impact strength, and hardness, reduced flammability
		ES powder in Polyamide composites [66]	Low-cost biocompatible filler material enhances flexural and tensile modulus.	Improved adsorption due to the porous structure and increased surface area
		ES powder and date palm fiber in Bio-epoxy composites [67]	Improves the mechanical properties of the composite	Improve tensile strength/modulus, flexural strength/modulus, and stiffness
	Bone scaffold	Sodium alginate/EW and ES powder/PCL [73]	ES improves mechanical stability; EW promotes cell adhesion	ES enhances bioactivity and compressive strength; EW improves biocompatibility
	Treatment of osteoporosis	ES powder [55]	Providing a natural source of calcium and other elements for bone health	Similar or better bioavailability than food grade purified calcium carbonate
Eggshell membrane	Skin grafts Wound healing	ES membrane and Polycaprolactone (PCL) [77]	Provides a scaffold for cell attachment and growth	Enhanced biocompatibility, antibacterial activity, mechanical strength, appropriate water uptake and degradation properties
Egg white	Tissue engineering	Raw EW/Gelatin [96]	Improves the scaffold’s bioactivity by providing bioactive proteins that promote cell growth and adhesion	Hydrocolloids with tunable viscosity, mechanical properties and high biocompatibility, supporting cell growth
		Raw EW/Gelatin/Carboxymethylcellulose [94]	Acts as a reinforcing biopolymer that enhances hydrogel stability	Self-adhesive, self-healing, and sensing capabilities with great biocompatibility
		Proteins derived from raw EW/PCL [111]	Improves hydrophilicity, enhances bioactivity, promotes cell proliferation/differentiation	Nanofibrous mats with enhanced water uptake, tensile strength, and cell interactions, suitable for tissue engineering scaffolds
		Raw EW/Alginate [112]	Enhances the gelation process and promotes cell attachment and growth	More affordable option with a slower degradation rate compared to Matrigel^®^
		EW/PVA film [113]	The EW provides bioactivity and improves cell adhesion by providing a rougher texture	Suitable mechanical strength, degradation rate, high swelling ratio, and optimal water vapor transmission
	Drug delivery	Raw EW/Chitosan hydrogel [99]	Enhances biodegradability; improves cellular interaction and adhesion	Biocompatible, antioxidant, anti-inflammatory, and antibacterial, accelerating healing of burn wounds
		Homogenized raw EW/Silk fibroin [114]	Enhances mechanical properties and flexibility; promotes cellular attachment and proliferation	A composite bioink with improved mechanical properties, controlled degradation, and enhanced cell compatibility
	Biosensors	Raw EW/Carbon Dots [101]	Provides a biodegradable and biocompatible matrix for integrating carbon dots	Compatible strength with skin; porous structure promoting cell infiltration; degradation aligned with hair follicles regeneration
		Raw EW hydrogel/CNT [6]	Provides excellent biocompatibility, shear thinning property for 3D printing	Highly stretchable and self-healing; 3D printable at room temperature, suitable for electronic sensors and humidity-responsive actuators
Egg yolk	Wound healing and Bone tissue engineering	Egg yolk derivatives such as IgY or phospholipids [105]	Promote wound healing and bone tissue engineering; provide antibodies for diagnostics	Contains various bioactive components, including lipids, proteins, vitamins, and minerals

## 3. Summary and Future Prospectives

In conclusion, the egg is an abundant, low-cost biomaterial that has demonstrated potential across a wide range of biomedical applications. Its unique properties, such as biocompatibility, biodegradability, gelling capability, and the presence of bioactive components like lysozyme, ovalbumin, and lgY, contribute to its versatility in areas including drug delivery, tissue engineering, wound healing and diagnostics. For instance, the calcium carbonate content of eggshells makes them an ideal candidate for bone regeneration, while the gelation properties of egg white enable its use in developing scaffolds for tissue engineering. Although knowledge gaps and challenges remain, ongoing research and development are actively addressing these issues, advancing the potential for broader clinical and commercial use of egg-based biomaterials in the future.

The future potential of eggs in biomedical engineering is vast, with ongoing research uncovering new and innovative applications. One promising area is the development of 3D in vitro platforms, where egg white serves as a versatile material for constructing three-dimensional tissue models and drug-delivery systems. Its natural transparency allows for real-time visualization of cell growth and behavior [82]. Additionally, egg-derived biomaterials have demonstrated the ability to stimulate angiogenesis and promote host tissue integration, thereby accelerating wound healing [115]. Coating medical implants and devices with lysozyme extracted from egg white is a potential strategy for preventing bacterial infections. Studies have shown that thermal and chemical treatments can further enhance lysozyme’s antibacterial efficacy against specific pathogens [12]. Furthermore, recent advances in genetic engineering have enabled the production of genetically modified hens that lay eggs enriched with therapeutic proteins and peptides, opening new possibilities for biopharmaceutical manufacturing and personalized medicine [116].

Despite growing interest in the biomedical applications of eggs, several research gaps must be addressed to fully harness their potential. One major challenge is standardization. The natural variability in egg composition calls for the development of standardized processing protocols to ensure reproducibility in both research and clinical applications. This includes optimizing conditions for processing egg white into hydrogels, such as pH, temperature, crosslinker types, and incorporation of materials like alginate or gelatin. Another critical area is immunogenicity. While egg-derived components are generally biocompatible, their potential to trigger immune responses needs to be thoroughly evaluated, particularly for long-term and in vivo applications. Furthermore, scalable production methods must be developed to support commercial translation, ensuring consistent quality, availability and affordability. The future research should prioritize closing these gaps by developing standardized processing techniques, evaluating immunological safety, and conducting long-term efficacy studies on egg-based biomaterials.
